# Disaster Exposure and Insomnia Severity During 7⋅20 Flood in Henan: The Moderated Mediation Model

**DOI:** 10.1002/pchj.70020

**Published:** 2025-07-11

**Authors:** Minqi Yang, Meimei Chu, Ruobing Cao, Chunyu Qu, Hanxiao Guo, Qian Zhou, Hanshuo Zhang, Jinlu He, Wenxuan Li, Jingjing Gu, Guofu Zhou

**Affiliations:** ^1^ Lab of Light and Physio‐Psychological Health National Center for International Research on Green Optoelectronics, South China Normal University Guangzhou China; ^2^ School of Education, Zhengzhou University Zhengzhou China; ^3^ Department Educational Sciences School of Social Sciences and Technology, Technical University of Munich Munich Germany; ^4^ Center for Mental Health Education Faculty of Psychology, Southwest University Chongqing China; ^5^ National Center for International Research on Green Optoelectronics South China Normal University Guangzhou China

**Keywords:** catastrophizing, dark triad, insomnia severity, natural disaster exposure

## Abstract

Natural disaster exposure is considered to be one of the risk factors for mental health. We investigated whether natural disaster exposure was associated with insomnia severity and the roles of catastrophizing and dark personalities in the association. The current study, using data collected from 1526 participants (27.50 ± 15.49 years old, 40.4% male), was conducted within 2 weeks after the 7⋅20 flood in Henan, China. Results showed that natural disaster exposure was significantly positively associated with insomnia severity, catastrophizing partially mediated the association between natural disaster exposure and insomnia severity, and the Dark Triad played moderating roles in the mediation model. Specifically, higher levels of the Dark Triad, Machiavellianism, and psychopathy weakened the negative link between disaster exposure and catastrophizing; whereas a higher level of narcissism exacerbated the relationships between natural disaster exposure and catastrophizing, and between natural disaster exposure and insomnia severity in the mediation model. The present results may provide important practical implications: the preventions and interventions that target the change of Dark Triad traits and the mitigation of catastrophizing could potentially be more effective in counteracting the development of sleep issues following exposure to floods.

## 1 Introduction

### 1.1 The Relation Between Disaster Exposure and Insomnia Severity

The July 20 Heavy flood (2021) in Henan province, China (7⋅20 flood), destroyed many shops and roads and killed more than 300 people. This natural disaster happened so suddenly and uncontrollably that some individuals got hurt or died directly, and some were trapped at home without electricity, water, and enough food, which brought not only huge economic losses to society but also a huge spiritual hit to people who were exposed to this natural disaster (Freedy et al. 1993).

Disaster exposure has a detrimental effect on psychological health (Nasri et al. 2020), including insomnia (Belleville et al. 2019; Ikeda et al. 2019). Insomnia refers to the difficulty with sleep. Its core symptoms are long sleep latency and frequent awakenings (Sateia et al. 2000). The associations between disaster exposure and sleep problems have already been proven. For example, traumatic events or disaster exposure could induce sleep disturbances and insomnia (Lavie 2001; Belleville et al. 2019), and the level of disaster exposure is significantly associated with insomnia (Geng et al. 2019; Ikeda et al. 2019). A systematic review has suggested that sleep disturbances, assessed one to ten months following the fires, were highly prevalent in wildfire survivors, with insomnia being the most prevalent sleep disturbance reported (Isaac et al. 2021). Thus, we hypothesized natural disaster exposure would be significantly and positively associated with insomnia severity (H1).

### 1.2 Catastrophizing as a Mediator

Exposure to natural disaster or negative life events may not be a necessary and sufficient condition for adverse outcomes such as insomnia. Even though studies indicate there is an association between disaster exposure and insomnia severity, few studies paid attention to the mediating role of the maladaptive cognitive emotion regulation strategy, such as catastrophizing. Catastrophizing, which refers to thoughts that emphasize the horror of what one has gone through (Ursu and Mairean 2022), is one experiencing a relatively minor negative event and imagining disasters resulting from this one event (Vasey and Borkovec 1992).

Within the framework of rational emotive behavior therapy (REBT), A. Ellis (1962, 1985) described “catastrophizing” as the tendency to magnify a perceived threat and overestimate the seriousness of its potential consequences. When a person thinks catastrophically, he or she usually looks at the adverse outcome of an event and decides that if that outcome were to happen, everything would be a disaster. In this process, bad emotions are infinitely amplified by the “self” which may eventually affect our normal life. Everyone engages in catastrophic thinking from time to time, which comes from anxiety and can lead to greater anxiety (Kendall and Ingram 1987). In addition, Peterson et al. (1998) have revealed that catastrophizing mediated the link between ways of explaining bad events and poor health. Consistently, some studies have demonstrated that negative life events could predict depression via negative cognitive emotion regulation strategy (Jiao et al. 2010; Wei and Zhang 2008). Thus, we hypothesized that the association between natural disaster exposure and insomnia severity could be mediated by catastrophizing (H2).

### 1.3 The Dark Triad and Its Moderation Effect

Not all individuals are equally impacted by disaster exposure and catastrophizing. Just as the researchers posited, the symptoms of psychopathology can vary depending upon an individual's personality traits (Widiger 2011; Papageorgiou et al. 2019). In the current study, we explored the moderating effects of the “dark side” of personality, Dark Triad, which consists of three distinct but overlapping negative personality traits: Machiavellianism, psychopathy, and narcissism (Jonason et al. 2013). Machiavellianism is characterized by being manipulative and strategic, psychopathy is known for lack of self‐control and deficits in affect, and narcissism refers to admiration‐seeking, superiority over others, and an egocentric attitude (Del Gaizo and Falkenbach 2008). Although the Dark Triad components share an anti‐social nature, lower agreeableness, emotional coldness, and self‐promotion (Paulhus and Williams 2002), they are heterogeneous by nature, comprising aspects of insecurity, vulnerability, anxiety, and impulsivity (Sabouri et al. 2016).

Studies have showed that individuals with a high level of Dark Triad have fewer prosocial behaviors and adopt more negative coping styles when they experience stressful life events, such as pessimism or disappointment (Birkás et al. 2016; Zhou and Zhang 2022; Qin and Gan 2024). Besides, due to their extreme level of insecurity, fear, and anxiety, individuals with Dark Triad experience negative outcomes more often than others, find it hard to trust others, and feel that others are always in search of opportunities to take advantage of them, which makes such individuals perceive their surroundings negatively (Lata and Chaudhary 2021). Due to their cynical view of human nature, people with Dark Triad perceive any ambiguous stimuli or behavior as a threat to themselves (Furnham et al. 2013). Thus, when individuals with Dark Triad are exposed to natural disasters, they would be more likely to carry a negative perception of themselves, others, and their surroundings, and adopt negative coping strategies, such as catastrophizing.

The associations between insomnia severity and disaster exposure, and between insomnia severity and catastrophizing may also be intensified by the Dark Triad. The vulnerability‐stress model (VSM) presumes that all people have some degree of vulnerability and its triggers, which depend on the interaction between vulnerability and stress (Monroe and Hadjiyannakis 2002). The VSM hints that the dark triad as a vulnerability factor would explain the occurrence of internalized or externalized problems in conjunction with stressful life events, stressful situations, or disasters (Monroe and Hadjiyannakis 2002). A recent study has revealed that, relative to the low‐level dark triad, the high‐level dark triad amplifies the detrimental impact of life events on nonsuicidal self‐injury (NSSI), one outcome of the internalized and externalized problems, especially when individuals are exposed to more negative life events (Gao et al. 2020).

Moreover, some empirical studies have examined the effect of Dark Triad on sleep‐related issues. For instance, individuals with high Dark Triad were inclined to stay up late and thus could be called as “creatures of night” or “night owls” (Jonason et al. 2013), which is related to lower sleep quality (Zhu et al. 2022). Besides, researchers demonstrated Dark Triad is a predictor of poor sleep quality, since they believed that the poor sleep quality may be caused by anxiety and low self‐esteem, the important characteristics of Machiavellianism and secondary psychopathy (Akram et al. 2018; Ali and Chamorro‐Premuzic 2010; Yang et al. 2019), and they also believed that negative cognitive‐emotional processes tend to cause sleep‐related problems (Sabouri et al. 2016). Coincidentally, Dark Triad is known for the deficiency in emotional stability (Birkás et al. 2016; Zeigler‐Hill and Vonk 2015) and emotional control (Birkás et al. 2016). In addition, according to the risk‐buffering hypothesis, favorable individual characteristics could buffer the negative effects of some risk factors on mental health (Luthar et al. 2015). Dark triad, as unfavorable individual characteristics, is supposed to exacerbate the negative effects of the risk factors. According to Life History Strategy Theory (LHT), which describes differences in the amount of bioenergetic and material resources allocated for somatic effort and reproductive effort (Jonason et al. 2010; Wilson 1975), individuals with slow Life History Strategies are willing to adopt protective interventions (Ellis et al. 2009; Chang et al. 2021), thus less likely to get negative outcomes such as internalizing problems (Chang et al. 2021). One study has showed slow Life History Strategies could moderate the link between childhood adversities and increased internalizing problems during the COVID‐19 pandemic (Chang et al. 2021). In order to gain more reproductive and living resources, individuals high in Dark Triad apply a fast life strategy exhibited by short‐term mating, risk‐taking tendency, and indifference to social morality (Jonason et al. 2010; Jonason and Tost 2010; McDonald et al. 2012). Additionally, Dark Triad is correlated with negative mental health outcomes (Gómez‐Leal et al. 2019; Shen 2023). Based on the above literature, we proposed that Dark Triad would negatively moderate the links among natural disasters, catastrophizing, and insomnia severity (H3).

### 1.4 The Present Study

**FIGURE 1:**
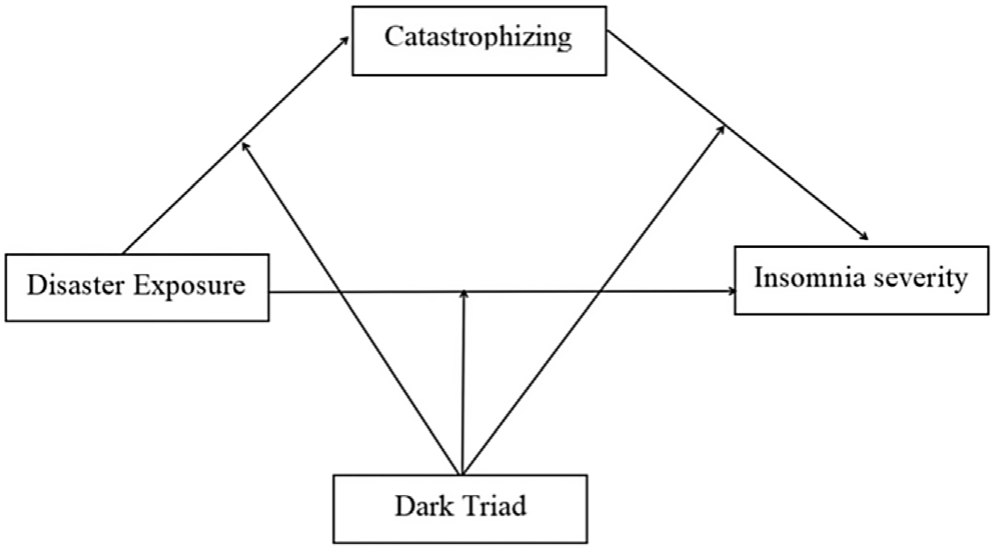
The hypothesized model.

The current study aimed to explore the relationships between disaster exposure and insomnia severity and the roles of catastrophizing and the Dark Triad in the association in the context of the July 20 heavy flood (2021) in Henan, China. Particularly, we established a moderated mediation model to answer the questions: how and when does disaster exposure affect insomnia? Based on the theories and empirical studies aforementioned, we hypothesized that (1) disaster exposure would be positively associated with insomnia severity, (2) catastrophizing would mediate the association between disaster exposure and insomnia severity, and (3) the Dark Triad would negatively moderate all the paths in the mediation model (Figure 1). We attempted to provide suggestions about the prevention and intervention for sleep problems during and after the flood.

## 2 Materials and Methods

### 2.1 Participants

We recruited 1526 participants (27.50 ± 15.49 years old, 40.4% male), who were exposed to the July 20 Heavy flood (2021) in Henan province of China, to do an online survey within 2 weeks after the natural disaster. The amount of participants was sufficient since the sample size calculated by using G*power software (by selecting the linear multiple regression and setting the fitting parameters: *R* ^2^ = 0.04, *α* = 0.05, 1 − *β* = 0.95) was only 481. The study was approved by the Ethical Committee of Zhengzhou University and carried out in accordance with the approved guidelines and regulations. All participants, recruited on a voluntary basis, confirmed their compliance by written informed consent and were paid for the participation.

### 2.2 Measures

#### 2.2.1 Disaster Exposure

The level of disaster exposure was measured by 34‐item Disaster Exposure Questionnaire (Liu 2011), which was adapted from Disaster Exposure Index (Wu et al. 2002). The disaster exposure questionnaire includes three dimensions: illness/injury to self or others (24 items), subjective feeling of fear (8 items), and property losses (2 items). In the current study, we changed the word of “earthquake” into “flood” in the questionnaire. The illness/injury to self or others dimension contains three types: trapped, injured and death, such as “one of my friends got hurt when the flood happened.” Subjective feeling of fear includes 8 items, such as “I was afraid of myself being dead.” Property loss mainly assesses the damage of one's own house and working building, and participants will respond on 4‐likert scale, ranging from 1 (*not damaged at all*) to 4 (*completely damaged*). The total score of the questionnaire is the sum of each dimension score, with higher scores indicating the high level of disaster exposure. In the present study, Cronbach's *α* for the scale was 0.925.

#### 2.2.2 Catastrophizing

Catastrophizing was measured by the Catastrophizing subscale (4 items) of Cognitive emotion regulation questionnaire (Garnefski et al. 2001). Items are like “*I often think that what I have experienced is much worse than what others have experienced*”. Each item is rated on a 5‐point scale (1 = never, 5 = always). Higher scores indicate greater catastrophizing. In the present study, Cronbach's *α* for the subscale was 0.947.

#### 2.2.3 Insomnia Severity

Insomnia severity was measured by Athens Insomnia Scale (Chung et al. 2011), which consists of 8 items. Items are like “During the last two weeks, the total sleep quality.” Each item is rated on a 4‐point scale (0 = *no problem*, 3 = *severely affected*). Higher scores indicate worse sleep quality. The higher score represents higher level of insomnia severity. In the present study, Cronbach's *α* for the scale was 0.956.

#### 2.2.4 Dark Triad

Dark Triad was measured by the Chinese version of Dirty Dozen (Geng et al. 2015), which consists of 12 items and three dimensions: Machiavellianism, psychopathy and narcissism. Items are like “I am inclined to manipulate others to achieve my goal”. Each item is rated on a 7‐point scale (1 = *totally disagree*, 7 = *totally agree*). Higher scores indicate higher level of Dark Triad personality. In the present study, Cronbach's *α* for the scale was 0.960. The Cronbach's *α* for Machiavellianism, psychopathy and narcissism were 0.970, 0.961, and 0.929, respectively.

### 2.3 Data Analysis

In the present study, correlations, means, and standardized deviations of all the study variables were examined through the use of SPSS 21.0. Then, we used the PROCESS macro Model 4 to test the mediating role of catastrophizing (Hayes 2013), and used Model 59 to test the moderating role of the Dark Triad in the mediation model.

## 3 Results

### 3.1 Preliminary Analyzes

Means, standard deviations, and correlations for all the study variables are presented in Tables 1 and 2. Pearson's correlation analysis results showed that all the study variables were significantly and positively correlated with each other.

**TABLE 1:** Means, standards, maximum and minimum values of study variables.

	*M*	SD	MIN	MAX
Insomnia severity	8.704	6.989	0.000	24.000
Disaster exposure	19.263	6.235	9.000	40.000
Catastrophizing	12.050	4.303	4.000	20.000
Dark Triad	45.024	19.539	12.000	84.000
Narcissism	18.197	6.195	4.000	28.000
Psychopathy	13.374	7.822	4.000	28.000
Machiavellianism	13.452	7.891	4.000	28.000

**TABLE 2:** Correlations deviations of study variables and demographic variables.

	1	2	3	4	5	6	7	8	9	10	11	12	13	14
1 Insomnia severity	1													
2 Disaster exposure	0.380**	1												
3 Catastrophizing	0.492**	0.237**	1											
4 Dark Triad	0.560**	0.341**	0.728**	1										
5 Narcissism	0.284**	0.122**	0.521**	0.755**	1									
6 Psychopathy	0.600**	0.386**	0.709**	0.940**	0.528**	1								
7 Machiavellianism	0.568**	0.365**	0.691**	0.951**	0.561**	0.923**	1							
8 Gender	0.213**	0.267**	0.169**	0.232**	0.054*	0.274**	0.260**	1						
9 Age	−0.009	−0.046	−0.075**	−0.133**	−0.173**	−0.087**	−0.106**	−0.055*	1					
10 Degree of education	−0.291**	−0.200**	−0.201**	−0.203**	0.004	−0.276**	−0.233**	−0.086**	−0.189**	1				
11 Occupation A	0.158**	0.130**	0.089**	0.085**	−0.037	0.124**	0.116**	0.046	0.421**	−0.204**	1			
12 Occupation B	0.127**	0.131**	0.109**	0.161**	0.075**	0.172**	0.170**	0.101**	−0.119**	−0.203**	−0.362**	1		
13 Occupation C	−0.176**	−0.127**	−0.102**	−0.159**	−0.049	−0.173**	−0.184**	−0.090**	−0.152**	0.211**	−0.289**	−0.186**	1	
14 Marital status A	0.162**	0.195**	0.108**	0.087**	−0.050*	0.137**	0.118**	0.038	0.534**	−0.297**	0.471**	−0.065*	−0.223**	1
15 Marital status B	0.139**	0.109**	0.108**	0.113**	0.068**	0.117**	0.110**	0.083**	0.104**	−0.120**	0.085**	0.024	−0.076**	0.195**

*Note*: Gender is coded as a dummy variable, represented by a “1” for males and a “0” for females. As for the education level, it was treated as continuous variable with “1” representing primary school level and below, “2” junior high school level, “3” senior high school level, “4” junior college level, “5” undergraduate level, “6” master's level, and “7” doctoral level. Occupation, divided into four categories: students (coded 1), professional technicians or administrative personnel (coded 2), workers or service personnel (coded 3), and other jobs (coded 4), was transformed into dummy variable in data analysis. In occupation A, student was coded as 0, and the others was 1; In occupation B, professional and technical personnel or administrative personnel was coded as 0, and the others were 1; In occupation C, worker or service worker was coded as 0, and the others were 1. The marital status was divided into three categories: “Unmarried”(coded 1), “Married” (coded 2) and “Divorced” (coded 3). In data analysis, these categories were transformed into dummy variables for processing. Marital Status A, where “Unmarried” was coded as 0 and the other two statuses as 1, and Marital Status B, where “Divorced” was coded as 1 and the other two statuses as 0. The covariates were same in Tables 3, 4, 5.

* *p* < 0.05.

** *p* < 0.01.

### 3.2 Testing for the Mediation Model

Model 4 from the SPSS macro PROCESS was used to test the mediation effect with catastrophizing as a mediator, and gender, age, education level, marital status, and occupation were taken into account as covariates, with marital status and occupation being transformed into dummy variables. The results showed that when catastrophizing was included, disaster exposure was positively associated with catastrophizing (*β* = 0.134, *p* < 0.001), which in turn was positively associated with insomnia severity (*β* = 0.380, *p* < 0.001) (Table 3).

**TABLE 3:** Testing the mediation effect.

Predictors	*t*	Catastrophizing			*t*	Insomnia severity		
		*β*	LLCI	ULCI		*β*	LLCI	ULCI
Disaster exposure	5.079***	0.134	0.082	0.186	9.600***	0.219	0.174	0.264
Catastrophizing					17.255***	0.380	0.336	0.423
Gender	3.670***	0.189	0.088	0.289	2.838**	0.125	0.039	0.212
Age	−5.579***	−0.167	−0.226	−0.108	−1.259	−0.033	−0.084	0.018
Education level	−4.998***	−0.133	−0.186	−0.081	−5.790***	−0.133	−0.179	−0.088
Occupation A	2.808**	0.190	0.057	0.322	2.669**	0.155	0.041	0.268
Occupation B	2.388*	0.175	0.031	0.319	1.510	0.095	−0.028	0.219
Occupation C	−0.501	−0.040	−0.197	0.117	−2.124*	−0.145	−0.279	−0.011
Marital status A	2.356*	0.152	0.026	0.279	0.073	0.004	−0.105	0.113
Marital status B	2.494*	0.396	0.085	0.707	2.110*	0.288	0.020	0.555

* *p* < 0.05.

** *p* < 0.01.

*** *p* < 0.001.

The indirect effect of catastrophizing was 0.051 (95% CI [0.031, 0.072]) with the direct effect being 0.219 (95% CI [0.174, 0.264]) (Table 4), which indicated that catastrophizing partially mediated the relationship between disaster exposure and insomnia severity.

**TABLE 4:** The direct effect and indirect effect in the mediation model.

	Effect	SE	LLCI 95%	ULCI 95%	Relative effect values
Direct effect	0.219***	0.023	0.174	0.264	81.111%
Indirect effect	0.051***	0.011	0.031	0.072	18.889%
Overall effect	0.270***	0.025	0.221	0.318	

*** *p* < 0.001.

### 3.3 Testing for the Moderated Mediation Model

Model 59 from macro PROCESS was applied to test for the proposed moderated mediation model with catastrophizing as a mediator and Dark Triad (psychopathy, narcissism, Machiavellianism) as moderators, and gender, age, education level, and marital status were taken into account as covariates. The results showed that the interaction of catastrophizing and narcissism was significant (*β* = 0.044, *p* < 0.01) (Table 5). These findings indicated that narcissism moderated the association between disaster exposure and insomnia severity as well as the relationship between catastrophizing and insomnia severity. Additionally, the results indicate that there is a significant interaction between disaster exposure and the Dark Triad (Machiavellianism, psychopathy and narcissism), suggesting that the Dark Triad moderates the relationship between disaster exposure and catastrophizing.

**TABLE 5:** Moderated mediation analyzes.

Predictor	Outcome: catastrophizing				Outcome: insomnia severity			
	*β*	*t*	LLCI	ULCI	*β*	*t*	LLCI	ULCI
Disaster exposure	0.134	5.079***	0.082	0.186	0.219	9.600***	0.174	0.264
Predictor								
Disaster exposure	−0.049	−2.351*	−0.090	−0.008	0.164	6.828***	0.117	0.211
Catastrophizing					0.166	5.610***	0.108	0.224
Dark Triad (moderator)	0.727	37.103***	0.689	0.766	0.323	10.024***	0.260	0.386
**Disaster exposure × Dark Triad**	**0.060**	**2.871** **	**0.019**	**0.101**	0.034	1.401	−0.013	0.081
Catastrophizing× Dark Triad					0.021	1.108	−0.016	0.057
Predictor								
Disaster exposure	0.077	3.394**	0.033	0.122	0.214	9.399***	0.170	0.259
Catastrophizing					0.328	12.679***	0.278	0.379
Narcissism (moderator)	0.510	23.871***	0.468	0.552	0.085	3.380**	0.036	0.135
**Disaster exposure × Narcissism**	**0.116**	**4.751** ***	**0.068**	**0.164**	**0.055**	**2.243** *	**0.007**	**0.104**
**Catastrophizing × Narcissism**					**0.045**	**2.584** **	**0.011**	**0.080**
Predictor								
Disaster exposure	−0.060	−2.637**	−0.104	−0.015	0.154	6.295***	0.106	0.202
Catastrophizing					0.183	6.500***	0.127	0.238
Machiavellianism (moderator)	0.698	32.931***	0.657	0.740	0.329	10.426***	0.267	0.390
**Disaster exposure × Machiavellianism**	**0.081**	**3.550** ***	**0.036**	**0.125**	0.037	1.510	−0.011	0.086
Catastrophizing × Machiavellianism	−0.060				0.008	0.411	−0.031	0.047
Predictor								
Disaster exposure	−0.087	−3.893***	−0.131	−0.043	0.138	5.565***	0.089	0.186
Catastrophizing					0.143	5.006***	0.087	0.200
Psychopathy (moderator)	0.734	34.981***	0.693	0.775	0.386	11.763***	0.322	0.451
**Disaster exposure × Psychopathy**	0.**097**	**4.292** ***	**0.052**	0.**141**	0.040	1.620	−0.008	0.089
Catastrophizing × Psychopathy					0.016	0.805	−0.023	0.055

* *p* < 0.05.

** *p* < 0.01.

*** *p* < 0.001.

**FIGURE 2:**
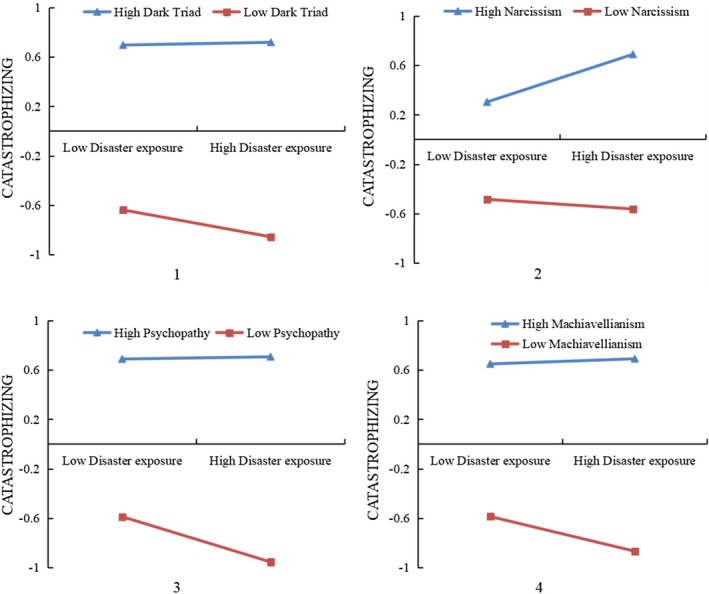
The moderating role of dark triad and its three components.

In order to further analyze the moderating effects of the Dark Triad and its components, their scores were divided into two groups separately according to 1 SD above and below the means, and simple effect analyzes were conducted. The results showed that, except the correlation was positive and stronger among individuals with lower narcissism (M + 1SD) (*β*_simple_ = 0.193, *p* < 0.001) than that among those with higher narcissism (M − 1SD) (*β*_simple_ = −0.039, *p* = 0.268) (Figure 2 (2)), the correlations between disaster exposure and catastrophizing were negative and stronger among individuals with lower Dark Triad and its three components. Specifically, the correlation among individuals with lower Dark Triad (M − 1SD) (*β*_simple_ = −0.109, *p* < 0.001) (Figure 2 (1)) than that among those with higher Dark Triad (M + 1SD) (*β*_simple_ = 0.011, *p* = 0.647); the correlation was stronger among individuals with lower psychopathy (M − 1SD) (*β*_simple_ = −0.184, *p* < 0.001) than that among those with higher psychopathy (M + 1SD) (*β*_simple_ = 0.009, *p* = 0.703) (Figure 2 (3)); the correlation was stronger among individuals with lower Machiavellianism (M − 1SD) (*β*_simple_ = −0.140, *p* < 0.001) than that among those with higher Machiavellianism (M + 1SD) (*β*_simple_ = 0.021, *p* = 0.398) (Figure 2 (4)).

**FIGURE 3:**
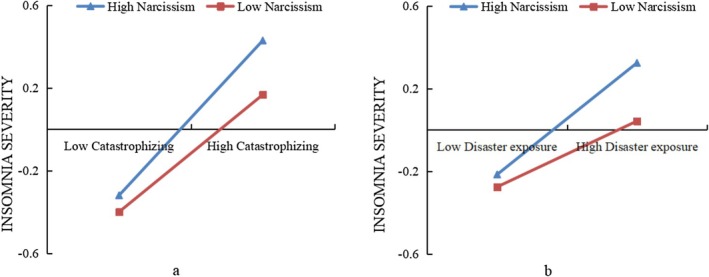
The moderating role of narcissism in the associations between catastrophizing and insomnia severity (a) and between disaster exposure and insomnia severity (b).

The results also showed that only narcissism bolstered the relationships between catastrophizing and insomnia severity, and between disaster exposure and insomnia severity. Specifically, the association between catastrophizing and insomnia severity among the individuals with higher narcissism (M + 1SD) (*β*_simple_ = 0.374, *p* < 0.001) was stronger than that among individuals with lower narcissism (M − 1SD) (*β*_simple_ = 0.283, *p* < 0.001) (Figure 3a), and the association between disaster exposure and insomnia severity among the individuals with higher narcissism (M + 1SD) (*β*_simple_ = 0.270, *p* < 0.001) was larger than that among the individuals with lower narcissism (M − 1SD) (*β*_simple_ = 0.159, *p* < 0.001) (Figure 3b).

## 4 Discussion

This study was the first to combine catastrophizing and the Dark Triad together to construct a moderated mediation model to explain how and when disaster exposure links to insomnia severity in the July 20 Heavy flood (2021) in Henan, China. The results revealed that catastrophizing mediated the association between disaster exposure and insomnia, the Dark Triad and its components moderated the path from disaster exposure to catastrophizing, and only narcissism moderated the paths from catastrophizing and disaster exposure to insomnia severity in the mediating model.

The present study showed disaster exposure was significantly and positively associated with insomnia severity, which was in line with the previous studies showing that exposure to natural disasters predicts insomnia or sleep‐related problems (Khazaie et al. 2019; Sugiura et al. 2013). What's more, disaster exposure can be regarded as one of the stressors. Stressful events and daily stress create psychological and organic symptoms that impair quality of life and sleep (Cuadros et al. 2012; Zamani Sani et al. 2023). For instance, Pillai et al. (2014) cited that stress exposure is a critical predictor of insomnia onset. Morin et al. (2003) found that insomniacs rated both the impact of daily minor stressors and the intensity of major negative life events higher than good sleepers. In addition, Palagini et al. (2016) suggested that some people who have both immune system problems and symptoms of insomnia are more likely to perceive challenging events as stressful.

This study also revealed catastrophizing mediated the relationship between disaster exposure and insomnia severity, which is basically consistent with previous studies showing that negative cognitive emotion regulation strategy mediates the relations between negative life events and negative outcomes, such as depression (Jiao et al. 2010; Wei and Zhang 2008). Emotion regulation consists of “all the extrinsic and intrinsic processes responsible for monitoring, evaluating, and modifying emotional reactions, especially their intensive and temporal features” (Gross 1999; Thompson 1994). Cognitive emotion regulation can be considered part of this broader concept and refers to conscious and cognitive ways of dealing with the intake of emotionally arousing information (Garnefski et al. 2001; Thompson 1991). As a maladaptive cognitive emotion regulation strategy, catastrophizing refers to the idea of explicitly emphasizing the horror of an experience.

Based on the adaptability, maladaptive strategies such as self‐blame or catastrophizing are detrimental to mental health, in contrast to adaptive strategies such as positive reappraisal, which are related to better mental health (Balzarotti et al. 2016; Domínguez‐Sánchez et al. 2013; John and Gross 2004; Mérida‐López et al. 2019; Nyklíček et al. 2011; Schäfer et al. 2017). A key characteristic of insomnia is the presence of negatively toned cognitive activity, mostly in terms of worry and rumination, which are closely related to catastrophizing (Harvey 2002). Individuals who adopt catastrophizing are prone to overestimate the negative effects of negative life events such as natural disasters (Garnefski et al. 2001) and fall into negative outcomes such as anxiety, poor sleep quality, and depression (Socci et al. 2020). Thus, it is reasonable to regard that catastrophizing may mediate the relationship between disaster exposure and insomnia severity.

The results showed that Dark Triad and its components played moderating roles in the mediation model. Specifically, higher levels of Dark Triad, Machiavellianism, and psychopathy weakened the negative link between disaster exposure and catastrophizing; only higher narcissism exacerbated the positive links among natural disaster exposure and catastrophizing and insomnia severity in the mediation model.

As for the moderating effects in the path from disaster exposure to catastrophizing, the results were basically consistent with previous studies. For instance, Mojsa‐Kaja et al. (2021) showed that the inappropriate use of adaptive and maladaptive cognitive‐emotional regulation strategies could be detrimental to the mental health of individuals with higher levels of DT traits. The vulnerable narcissistic personality relates to increased affects from stressful circumstances (Gore and Widiger 2016), and people with higher narcissism and Machiavellianism have more maladaptive strategies (Gómez‐Leal et al. 2022). Individuals high in psychopathy tend to use less adaptive strategies (Gómez‐Leal et al. 2022) and have deficits in emotion regulation, which then serves to accentuate negatively toned cognitive activity (Palagini et al. 2016), such as catastrophizing. Besides, individuals with the Dark Triad (regarded as an indicator of fast life history strategies, according to LHT) are more likely to adopt non‐adaptive strategies (Jonason and Tost 2010; McDonald et al. 2012), such as catastrophizing, one of the maladaptive cognitive emotional strategies.

The results also showed that narcissism moderated the paths from disaster exposure and catastrophizing to insomnia severity. Specifically, the association between catastrophizing and insomnia severity (Figure 3a) and the association between disaster exposure and insomnia severity (Figure 3b) among individuals higher in narcissism were much stronger. The results could be interpreted by the risk‐buffering hypothesis (Luthar et al. 2015). Narcissism, as an unfavorable individual characteristic, may exacerbate the negative effects of the risk factors on mental health. The Dark Triad components are heterogeneous by nature, comprising aspects of insecurity, vulnerability, anxiety, and impulsivity (Sabouri et al. 2016). However, they share an anti‐social nature, lower agreeableness, emotional coldness, and self‐promotion (Paulhus and Williams 2002). It has been reported that people having any of these three negative personality traits experience negative outcomes more often than others, due to their extreme level of insecurity, fear, and anxiety (Lata and Chaudhary 2021). Some studies have shown that narcissistic people tend to be more prone to anxiety (Pincus et al. 2014; Besser and Priel 2010). This may be because people with higher narcissistic tendencies are more concerned with their physical appearance and social status (Pincus et al. 2014). In particular, vulnerable narcissists are more likely to suffer from internal anxiety and insecurity, have doubts about their own worth and abilities, and are more sensitive to the evaluations of others.

Additionally, previous studies have found that individuals with narcissism are positively associated with negative emotions (Bonfá‐Araujo et al. 2021; Gómez‐Leal et al. 2019), which are usually linked with poor sleep quality (Becker et al. 2015; Brand et al. 2015). Thus, we infer that high narcissism would amplify the links from insomnia severity to catastrophizing and to flood exposure. It is acknowledged that negative cognitive‐emotional processes tend to cause sleep‐related problems such as sleep disturbances and insomnia (Sabouri et al. 2016). Narcissistic people may engage in unhealthy self‐regulation strategies (Ronningstam 2016), which may lead to the development or exacerbation of mood disorders (Miller et al. 2007). Considering the above evidence, individuals with higher narcissism are more likely to feel more negative emotions, which will intensify the association between catastrophizing and insomnia severity.

## 5 Implications

Insomnia has been proved to produce detrimental influence on individuals' mental health (Chi et al. 2021) and academic engagement (Scotta et al. 2021). Understanding the factors that contribute to insomnia may help prevent and intervene in insomnia, which further improves mental health, quality of life, and long‐term health (Brown et al. 2015).

The current study established a moderated mediation model which revealed the relationships between disaster exposure and insomnia severity and the psychological mechanism under the context of the July 20 heavy flood (2021) in Henan, China. The present results may provide important practical implications that the prevention and interventions focusing on changing the personality and maladaptive cognitive emotional strategies may help offset the occurrence of sleep problems after flood exposure more effectively.

First, this study indicates that catastrophizing is one of the explanatory factors for how disaster exposure can lead to insomnia severity, which indicates that reducing the level of catastrophizing is helpful to reduce the level of insomnia. Specifically, the psychological counselors and therapists could work on the cognitive vulnerability (i.e., negative coping style) of the maladaptive population through Cognitive Behavior Therapy (CBT) to weaken the catastrophizing from natural disaster exposure to insomnia.

Besides, the present study revealed that higher levels of Dark Triad, Machiavellianism, and psychopathy weakened the negative link between disaster exposure and catastrophizing. It also suggested that higher narcissism exacerbated the positive links among natural disaster exposure, catastrophizing, and insomnia severity in the mediation model. The present study provided us with a new perspective to understand why some individuals will react differently to similar circumstances and cognitive emotional regulation strategies such as catastrophizing. Moreover, interventions for the sleep problems should also focus on changing the Dark Triad.

## 6 Limitations and Future Directions

As far as we know, this is one of the first studies that used a large sample and revealed how and when the disaster exposure affects the insomnia severity in the context of the July 20 heavy flood (2021) in Henan, China. The present results had important implications for the prevention and intervention of sleep problems during the natural disaster. However, there are several limitations of the current study that should be considered. Firstly, we conducted mediation analyzes employing cross‐sectional data that were supposed to be used to explore the correlational relationships among these variables rather than to make causal inferences. Thus, longitudinal studies are needed to verify the findings in the current study. Secondly, all data were collected through self‐reported online surveys, and not all possible participants had access to the device; thus, there may be participation bias and response bias (e.g., social desirability). Therefore, future studies could apply the combination of online and offline surveys, the combination of self‐report and other‐report, and add a social desirability questionnaire to reduce response bias. Thirdly, there are some other protective variables such as resilience and social support that may also play as underlying mechanisms between disaster exposure and insomnia severity. Future studies could include these variables.

## 7 Conclusions

The current study contributes to the literature by testing a moderated mediation model, which offers an elaborate understanding of the relationships between disaster exposure and insomnia severity. The results indicated that disaster exposure was a risk factor for insomnia. Besides, mediation analyzes revealed that catastrophizing could be one explanatory factor for why disaster exposure is related to insomnia severity. Moreover, moderated mediation analyzes indicated that the Dark Triad traits could moderate the links among disaster exposure, catastrophizing, and insomnia severity.

## Conflicts of Interest

The authors declare no conflicts of interest.

## Data Availability

The datasets generated during and/or analyzed during the current study are available from the corresponding author on reasonable request.
